# Assessing residual reasoning ability in overtly non-communicative patients using fMRI^☆^

**DOI:** 10.1016/j.nicl.2012.11.008

**Published:** 2012-11-30

**Authors:** Adam Hampshire, Beth L. Parkin, Rhodri Cusack, Davinia Fernández Espejo, Judith Allanson, Evelyn Kamau, John D. Pickard, Adrian M. Owen

**Affiliations:** a The Brain and Mind Institute, University of Western Ontario, London, Ontario, Canada; b The MRC Cognition and Brain Sciences Unit, Cambridge, UK; c Department of Clinical Neurosciences, University of Cambridge, Cambridge, UK

**Keywords:** Cognitive assessment, Vegetative state, Minimal conscious state, Reasoning, Frontal lobe function, Mental imagery

## Abstract

It is now well established that some patients who are diagnosed as being in a vegetative state or a minimally conscious state show reliable signs of volition that may only be detected by measuring neural responses. A pertinent question is whether these patients are also capable of logical thought. Here, we validate an fMRI paradigm that can detect the neural fingerprint of reasoning processes and moreover, can confirm whether a participant derives logical answers. We demonstrate the efficacy of this approach in a physically non-communicative patient who had been shown to engage in mental imagery in response to simple auditory instructions. Our results demonstrate that this individual retains a remarkable capacity for higher cognition, engaging in the reasoning task and deducing logical answers. We suggest that this approach is suitable for detecting residual reasoning ability using neural responses and could readily be adapted to assess other aspects of cognition.

## Highlights

► Hampshire et al. develop a paradigm for assessing reasoning ability using fMRI alone. ► Inferior frontal sulcus activation is used as a marker of engagement in reasoning. ► Visual and pre-motor activation is used to detect logically correct answers. ► An overtly non-responsive patient is able to solve grammatical reasoning problems. ► Frontal lobe sub-regions activate at distinct stages of the reasoning task.

## 1 Introduction

Recent advances in functional brain imaging have provided a unique window into the inner world of physically non-communicative patients. Of particular relevance to the current study, is the observation that some of these patients retain a capacity for volitional cognitive processes in the absence of volitional motor actions (Owen et al., 2006). More specifically, a proportion of patients with clinical diagnoses of vegetative state (VS), also known as ‘unresponsive wakefulness syndrome’ (Laureys et al., 2010), or minimally conscious state (MCS) reliably engage in mental imagery in response to simple cues, for example, imagining moving parts of their body (Cruse et al., 2011) or mentally navigating around a familiar environment (Owen and Coleman, 2007). These types of internalised response can be detected using functional magnetic resonance imaging (fMRI) (Owen et al., 2006) or electroencephalography (EEG) (Cruse et al., 2011) as they activate functionally specialised premotor and posterior brain regions. Moreover, they may even be used to communicate simple yes–no answers to auditory questions. Indeed, this approach, in which category specific imagery provides a basic means of real-time communication, has been validated for physically non-communicative patients using autobiographical questions, the answers to which could subsequently be verified by family members (Monti et al., 2010).

The advent of such methods raises an immediate issue regarding what questions should be asked of patients who cannot communicate using overt motor responses. More specifically, whilst the ability to perform the previously reported neuroimaging tasks requires some spared capacity for interpreting statements, maintaining relevant information, and focusing attention (Cruse et al., 2011; Monti et al., 2010), higher cognitive functions like reasoning have not been directly assessed. Yet, if a physically non-communicative patient were ever to be asked to make decisions regarding the course of their long-term maintenance or whether potentially aversive treatments should be administered, it would first be necessary to demonstrate that they were able to make logical inferences in response to complex questions. However, developing cognitive assessment tools that do not require motor responses is fraught with difficulties. Consequently, whilst there has been much recent interest in developing fast and accurate EEG and fMRI methods for communication (Allison et al., 2007; Cusack et al., 2012), we know of no examples of these devices being used to assess higher cognitive functions in patients. Furthermore, although results from the few examples that have sought to assess reasoning processes in controls provide useful reference points (Perego et al., 2011), they invariably depend on visual stimuli that would be inappropriate in VS and MCS patients, for whom a lack of reliable gaze control is a defining symptom.

The aim of the current study was to develop a simple fMRI method for assessing reasoning processes by examining functional brain activity alone. We adapted a verbal reasoning task that was known to be related to intelligence as measured by classical IQ testing (Baddeley, 1968) and that, based on recent imaging research (Hampshire et al., 2012), we predicted would recruit frontal-lobe networks that have been implicated in higher cognitive function. To avoid the confounding effects of impaired eye movement control, we used an auditory task with a novel imagery-based interface. This simple technique, in which the participant imagined the object that formed the logically correct answer, was used to determine whether participants reliably solved the verbal reasoning problems. By examining the finite impulse response (FIR) timecourse of the neural response, we defined regions of interest (ROIs) and optimal time windows for detecting cognitive processes at three distinct stages of the reasoning trials: these being 1) the processing of auditory stimuli within the temporal lobes, 2) the logical transformation of grammatically complex problems within the frontal lobes, and 3) mental imagery of the selected answer within category specific premotor and posterior brain areas. Using these functional–anatomical markers, we looked for signs of reasoning in a physically non-communicative patient who had a long-term diagnosis of VS, but whom we believed to be conscious based on assessment with a previously validated motor imagery paradigm (Boly et al., 2007).

## 2 Methods

### 2.1 Paradigm

**Fig. 1:**
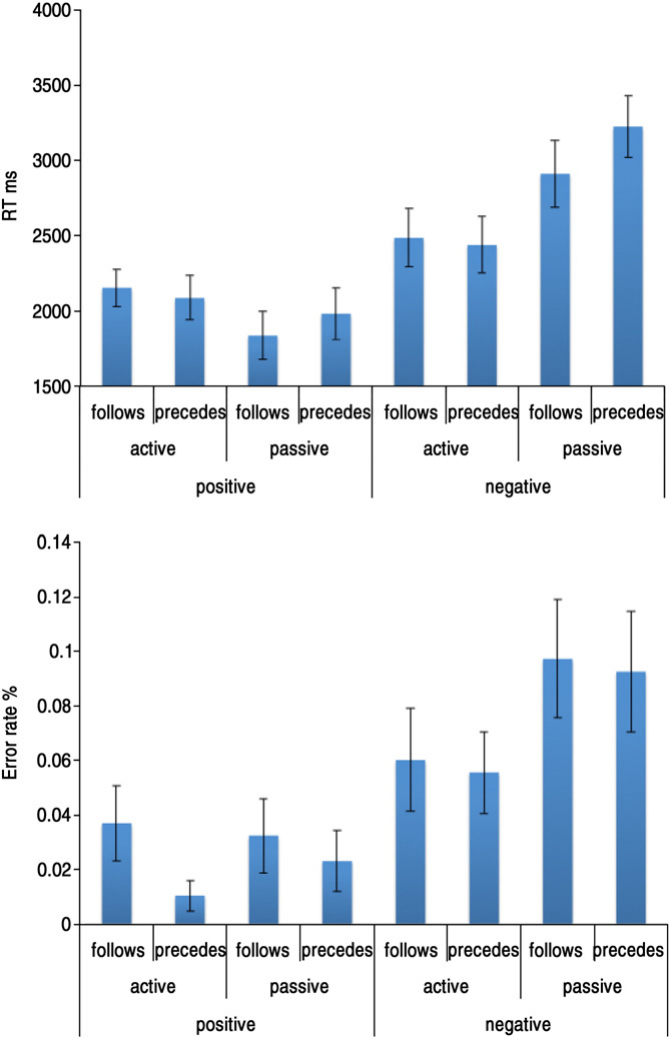
The behavioural pilot study (N = 21). At the group level, the participants were able to perform all trials significantly above chance (all 8 statements p < 0.001). At the individual subject level, there were two outliers (> 3 standard deviations from the mean), who performed at around chance (51.04% and 56.25%). Another participant performed poorly on the more difficult levels (4, 6, 7 and 8). Top — Response time data for each of the 8 verbal reasoning problems with 2 outliers removed. 3-Way repeated measures analysis of variance (ANOVA) showed significant main effects of active vs. passive voice (F_(1,18)_ = 10.19, p < 0.005) and positive vs. negative statements (F_(1,18)_ = 87.12, p < 0.001) and a sub threshold trend for the word follows vs. precedes ((F_(1,18)_ = 4.26, p = 0.054). There was also a significant interaction of active/passive ∗ positive/negative (F_(1,18)_ = 45.20, p < 0.001). Bottom — Error rates showed main effects of active vs. passive voice (F_(1,18)_ = 4.70 p < 0.05) and of positive vs. negative statements (F_(1,18)_ = 16.79 p < 0.001), but not of the word follows vs. precedes (F_(1,18)_ = 0.48 p = 0.50).

The verbal reasoning paradigm was adapted from Alan Baddeley's 3-minute grammatical transformation task (Baddeley, 1968). Participants were presented with statements describing the ordering of two objects, a face and a house (Table 1). They were instructed to deduce which of the objects was in front and to visualise that object in their mind. For example, if they heard the statement ‘the face is not followed by the house’ the correct answer would be ‘house’. Conversely if they heard ‘the face precedes the house’ the correct answer would be ‘face’. The statements were grouped into four levels of demand (L1–4) according to difficulty as calculated in a behavioural pilot study (Fig. 1). There were also cued trials, in which the participant was simply instructed to imagine one object or the other. It was intended that the top-down modulation of activation within category sensitive visual processing areas (Epstein and Kanwisher, 1998; Kanwisher et al., 1997; O'Craven and Kanwisher, 2000) during mental imagery, would provide a method for detecting the participant response and that such responses to simple cued trials would provide a within-task method for functionally localising these areas.

The statements (Table 1) were structured in predefined pseudo-randomised blocks. At the beginning of each block, the participant heard the command ‘imagine the object which is in front’ followed by four consecutive reasoning trials. On each trial, the participant heard a statement followed by a delay period. During the delay period, they were required to solve the problem as quickly as possible and to focus their mind on imagining the object that formed the correct answer. At the end of the delay period they heard the instruction ‘now rest’ subsequent to which they were required to relax until the start of the next trial. After four reasoning trials, there were two cued imagery trials, which had the same structure, but at the start of which the participant was simply instructed to either ‘imagine a face’ or ‘imagine a house’ and for which there was, therefore, minimal reasoning demand. The order in which the trials were presented was counterbalanced to ensure, that each of the 8 statements appeared once every 2 blocks, the answer ‘face’ or ‘house’ was balanced across each of the eight grammatical statements, and the probability of having two consecutive trials with the same or a different answer was also balanced.

### 2.2 Procedure

Prior to entering the scanner, the participants undertook a short practice session that involved listening to a pre-recorded verbal explanation of the task, followed by a description of how best to imagine a face and a house and practice examples. In the control study, there were 2 ∗ 10 minute experimental sessions that were designed to identify the optimal task design by varying the trade-off between the length of time allowed for imagery and the total number of trials that were attempted. One session used a slow design with four blocks. The participant had 10 s to work out and imagine the correct answer after each problem followed by 10 s of rest. The other session used a faster design that consisted of 6 blocks. The participant had 5 s to work out and imagine the correct answer followed by 5 s of rest. The order in which the fast and the slow sessions were undertaken was counterbalanced across participants. In controls, the slow design proved to be more reliable at the single subject level. (More specifically, of the 15 participants that showed significant activation for cued house > face imagery in one or more PPA ROIs, 80% also showed activation in the reasoning house–face contrast in the slow design compared with just 47% in the fast design). Consequently, only the slow design was applied in the individual patient case study.

### 2.3 Neuroimaging

fMRI data were collected using the standard 2 second fMRI protocol on a Siemens 3 T Tim Trio scanner in the Wolfson Brain Imaging Centre at Addenbrookes Hospital, Cambridge, UK. The data were pre-processed and analysed in SPM5 (Statistical Parametric Mapping, Welcome Department of Imaging Neuroscience, London, UK). Prior to analysis, data were slice timing and motion corrected, spatially normalized to the standard Montreal Neurological Institute template and spatially smoothed with an 8 mm full width at half maximum Gaussian kernel. The data were high-passed filtered (cutoff period = 180 s) to remove low frequency drifts in the MRI signal. Fixed effects analyses were carried out at the individual-subject level using general linear modelling. The combined reasoning and imagery period for each trial was defined from the time at which the auditory presentation of the grammatical statement or imagery cue ended. For each experimental run, there were a total of 10 sets of onset times corresponding to the imagery of either faces or houses in response to grammatical reasoning problems at each of the four levels of difficulty and simple cues. The onset times were modelled using finite impulse response functions consisting of 10 ∗ 2 second contiguous boxcar regressors that accounted for the 20 second period of time subsequent to the presentation of the reasoning statement or imagery cue. Whole brain maps depicting contrasts between beta weights for the model regressors were calculated at the individual subject level and these were exported for group level random effects analyses. Group level analyses were carried out unconstrained within the whole brain in SPM5. Focused regions of interest (ROIs) analyses were carried out with the MarsBaR toolbox, which calculates the average value from all voxels within a ROI. Analyses that included ROI as a factor were undertaken in SPSS.

### 2.4 Control participants

20 volunteers between the ages of 19 and 27 took part in the neuroimaging study. Participants spoke good English, were right handed, had good or corrected to good eyesight, normal hearing and no history of neurological or psychiatric illnesses.

### 2.5 Patient history

The patient was a 45-year-old male, who was admitted to hospital in January 2008 after being hit by a car. Shortly thereafter, he underwent a bilateral frontal decompressive craniectomy and evacuation of a left temporal haematoma. Structural MRI scans revealed extensive multicystic encephalomalacia in the frontal lobes and anterior temporal lobes, as well as damage to the left medial parietal lobe. He opened his eyes seven days after the accident, but subsequently showed no significant recovery. The patient was left with widespread brain damage, particularly in the anterior/medial frontal lobes and the anterior temporal lobes. He undertook behavioural assessments, MRI scanning sessions and electroencephalography (EEG) at Addenbrookes Hospital, Cambridge, UK. Behavioural assessment (Table 3) was undertaken on 11 separate days across 4 separate assessment weeks using the JFK Coma Recovery Scale (Giacino et al., 2004). At the time of his first assessment 18 months after his accident, he fulfilled all the criteria for the vegetative state but had a large skull defect. During his second admission 2.5 years after his accident, he demonstrated a portfolio of behaviours including flexion withdrawal responses. On occasion but not consistently, he showed some tracking in his left visual field. There were occasional small spontaneous movements of the toes and the right hand. On one occasion, the spontaneous movements appeared to increase after speaking to him but these did not stop upon spoken request. Behavioural examination was complicated by spasticity and anti-spastic drugs with sedative action (Supplemental Tables 1 & 2). The most positive behaviours were seen when the patient was lying flat, suggesting that he may have been susceptible to syndrome of the trephined. There was response to sound but no positive evidence of speech comprehension on functional MR or EEG (Owen et al., 2005). However, neuroimaging assessment with a previously validated motor imagery paradigm (Boly et al., 2007) revealed that on the day the current study was undertaken, he reliably engaged in category specific imagery in response to simple auditory instructions. Data for the present study were collected during this second admission. During his third admission 3 years post accident he may have shown eye movement to the right in response to verbal command, however, this responses could not be replicated. On the last assessment at the time of submission 4.5 years post accident, he has shown no further signs of recovery. This pattern of observations highlights the complications of motor assessment in such patients. This individual would be diagnosed as VS based on 9 of the 11 assessment days, but could be more accurately diagnosed as being minimally conscious based on the last day of session 2 and the second day of session 3. However, it was altogether unclear whether these movements were in fact generated in response to the tester, as they proved to be difficult to replicate. By contrast, functional neuroimaging on the day of testing revealed that whilst the patient did not make reliable motor responses on command, they did engage in category specific imagery in response to simple auditory instructions, and consequently, we would argue that they should be considered to have been physically non-communicative.

The patient was a 45-year-old male, who was admitted to hospital in January 2008 after being hit by a car. Shortly thereafter, he underwent a bilateral frontal decompressive craniectomy and evacuation of a left temporal haematoma. Structural MRI scans revealed extensive multicystic encephalomalacia in the frontal lobes and anterior temporal lobes, as well as damage to the left medial parietal lobe. He opened his eyes seven days after the accident, but subsequently showed no significant recovery. The patient was left with widespread brain damage, particularly in the anterior/medial frontal lobes and the anterior temporal lobes. He undertook behavioural assessments, MRI scanning sessions and electroencephalography (EEG) at Addenbrookes Hospital, Cambridge, UK. Behavioural assessment (Table 3) was undertaken on 11 separate days across 4 separate assessment weeks using the JFK Coma Recovery Scale (Giacino et al., 2004). At the time of his first assessment 18 months after his accident, he fulfilled all the criteria for the vegetative state but had a large skull defect. During his second admission 2.5 years after his accident, he demonstrated a portfolio of behaviours including flexion withdrawal responses. On occasion but not consistently, he showed some tracking in his left visual field. There were occasional small spontaneous movements of the toes and the right hand. On one occasion, the spontaneous movements appeared to increase after speaking to him but these did not stop upon spoken request. Behavioural examination was complicated by spasticity and anti-spastic drugs with sedative action (Supplemental Tables 1 & 2). The most positive behaviours were seen when the patient was lying flat, suggesting that he may have been susceptible to syndrome of the trephined. There was response to sound but no positive evidence of speech comprehension on functional MR or EEG (Owen et al., 2005). However, neuroimaging assessment with a previously validated motor imagery paradigm (Boly et al., 2007) revealed that on the day the current study was undertaken, he reliably engaged in category specific imagery in response to simple auditory instructions. Data for the present study were collected during this second admission. During his third admission 3 years post accident he may have shown eye movement to the right in response to verbal command, however, this responses could not be replicated. On the last assessment at the time of submission 4.5 years post accident, he has shown no further signs of recovery. This pattern of observations highlights the complications of motor assessment in such patients. This individual would be diagnosed as VS based on 9 of the 11 assessment days, but could be more accurately diagnosed as being minimally conscious based on the last day of session 2 and the second day of session 3. However, it was altogether unclear whether these movements were in fact generated in response to the tester, as they proved to be difficult to replicate. By contrast, functional neuroimaging on the day of testing revealed that whilst the patient did not make reliable motor responses on command, they did engage in category specific imagery in response to simple auditory instructions, and consequently, we would argue that they should be considered to have been physically non-communicative.

## 3 Neuroimaging study in controls

Markers of auditory, reasoning, and imagery processes were identified in controls by contrasting neural responses at different stages of the 20 second long finite impulse response (FIR) timecourse subsequent to stimulus delivery. Based on prior research, we expected to observe neural responses to these stages in the superior temporal lobes, the lateral frontal cortex and category sensitive posterior brain and premotor regions respectively.

### 3.1 Auditory processing

**Fig. 2:**
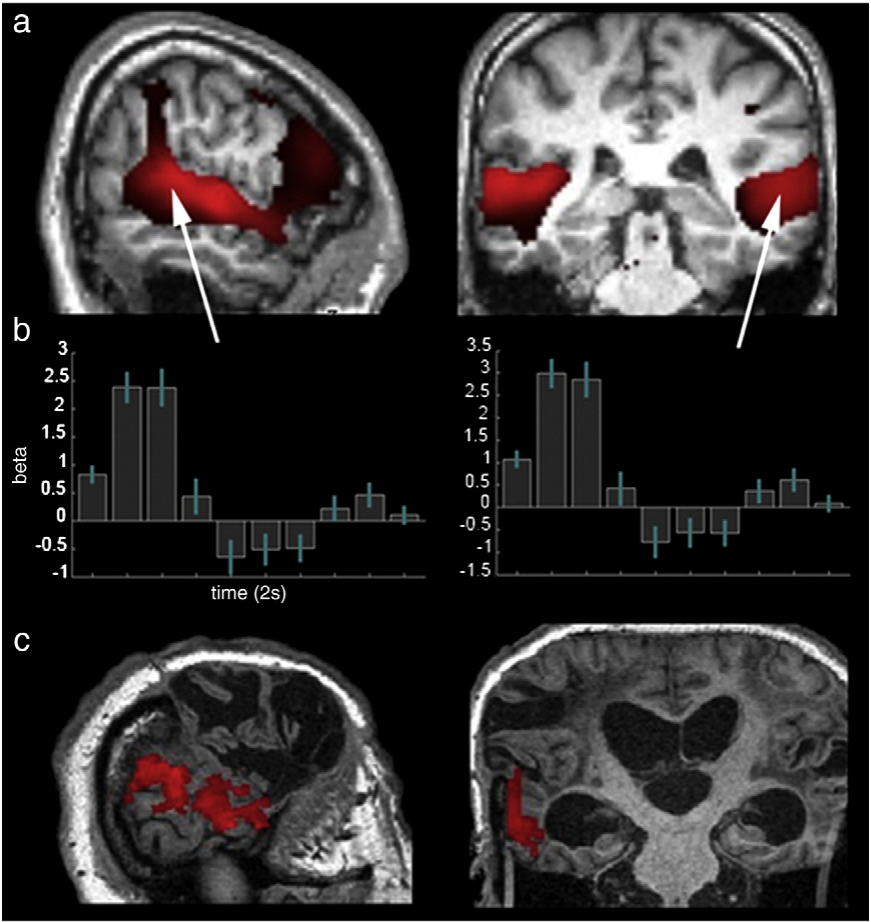
Temporal lobe activation subsequent to presentation of the auditory stimuli. a) In controls, the main effect of time rendered extensive activation subtending the superior temporal gyrus bilaterally (image rendered at p < 0.05 FDR corrected for the whole brain mass). b) The timecourse data from 5 mm spherical ROIs positioned at the peak activation coordinates within the temporal lobes. The BOLD response was strongest in the first three time bins (error bars report the 90% confidence interval. c) In the patient analysis, contrasting the first three time bins, where the temporal response was greatest in controls, vs. the rest of the timecourse, rendered significant activation in the left STL (image thresholded at t > 2.35 and FWE cluster corrected at p < 0.05).

**Fig. 3:**
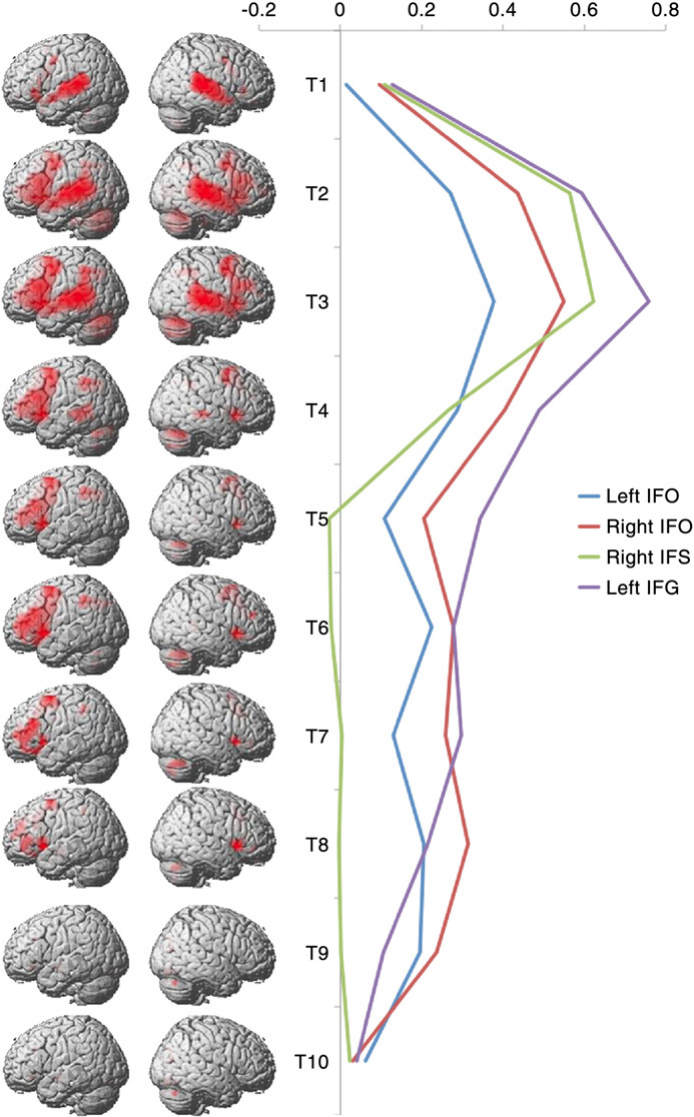
Average FIR timecourses from the reasoning trials. Left — Whole brain analyses for each of the 10 time bins, FDR corrected at p < 0.05 for the whole brain mass. Right — The bilateral IFO and left IFG ROIs showed sustained activation with a slight peak in the first half of the timecourse. The right IFS ROI showed a more pronounced peak and was selectively activated in the first half of the timecourse.

Whole-brain maps were generated, depicting mean activity at each of the 10 ∗ 2 second time bins subsequent to delivery of the reasoning problems. These data were analysed at the group level in SPM5 using a one-way repeated-measures analysis of variance (ANOVA) in which the condition was time (10). The positive main effect of condition (T contrast averaged across all 10 time bins) revealed extensive activations bilaterally within the posterior-superior temporal lobes (STL left x = − 60, y = − 22, z = 0 t = 5.22, right x = 46, y = − 32, z = − 2 t = 5.97, false discovery rate (FDR) whole brain corrected threshold p < 0.05). Significant regional activations were also evident in the insula/frontal operculum (IFO) bilaterally, focally in the right inferior frontal sulcus (IFS) and in the left inferior frontal gyrus (IFG). The main effect of time (Fig. 2a) was particularly strong within the STL (left x = − 56, y = − 22, z = 2, F = 32.65, p < 0.001 FDR corrected, right x = 62, y = − 20, z = 2, F = 37.39, p < 0.001 FDR corrected) and significant within the frontal lobes. Examination of the timecourse data, averaged across all voxels within 5 mm radius spheres of the peak STL coordinates, revealed strong activity that peaked in the second and third time bins and rapidly declined thereafter (Fig. 2b). When unconstrained whole brain analyses were carried out at each time bin using an FDR corrected threshold of p < 0.05 (Fig. 3), there was strong STL activation selectively at the earliest time points and weaker but more sustained frontal lobe activation. Based on these results, it was decided that STL activation during the first three time bins would provide the best marker of early auditory processing in the patient analysis.

### 3.2 Reasoning

**Fig. 4:**
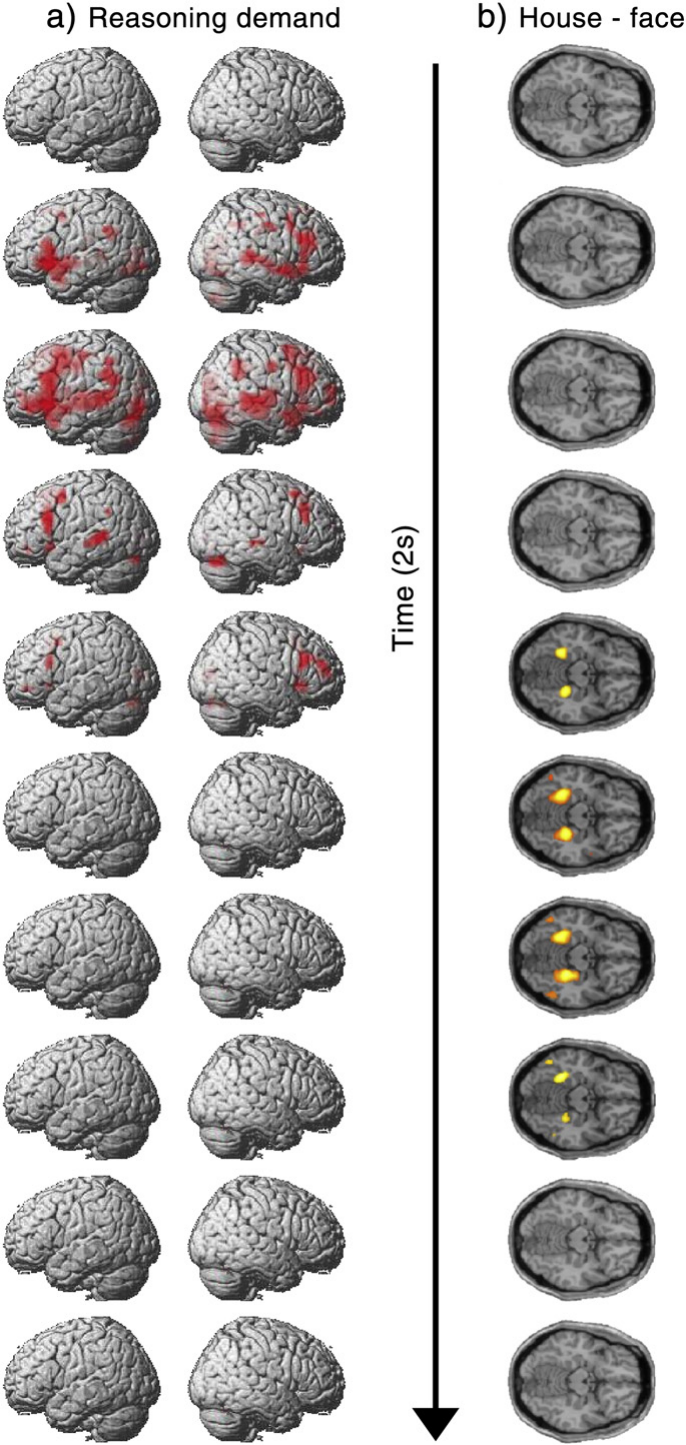
a) Whole brain analyses for the difficulty level contrast during reasoning trials at each of the 10 time bins. b) Whole brain analysis for the trials on which the correct answer was house minus those on which the correct answer was face at each of the 10 time bins. All results are presented with FDR correction at p < 0.05 for the whole brain mass.

10 mm spherical ROIs were defined centered on the peak activation coordinates from a prior study, in which the frontal lobes were fractionated into three functionally distinct networks using factor analyses (Hampshire et al., 2012). The first network included the IFO bilaterally and was associated with short-term memory (left x = − 42, y = 6, z = 0; right x = 45, y = 12, z = − 3). The second network included the left IFG and was associated with verbal processing (x = − 54, y = 33, z = 3). The third network included the right IFS and was associated with reasoning (x = 54, y = 36, z = 24). When the timecourse data were averaged across all reasoning trials (group-level model described in the previous section) and examined using a 4 ∗ 10 (ROI ∗ time) repeated measures ANOVA, there was a significant ROI ∗ time interaction (F_(27,513)_ = 3.41, p < 0.001) and significant main effects of ROI (F_(27,513)_ = 3.87 p = 0.014) and time (F_(9,171)_ = 7.39 p < 0.001). Examination of the timecourse data (Fig. 3) revealed that all frontal ROIs showed a peak in activity during the first half of the timecourse. However, the IFO and the left IFG ROIs showed sustained activation throughout the second half of the timecourse whereas the right IFS did not. The sensitivities of the frontal lobe ROIs to the overall level of reasoning demand were examined by calculating the weighted mean beta values across difficulty levels ((L1 ∗ − 2 + L2 ∗ − 1 + L3 ∗ 1 + L4 ∗ 2)), separately for each individual, for each time bin and for each ROI. The 4 ∗ 10 repeated measures ANOVA (ROI ∗ time) revealed a significant main effect of time (F_(9,171)_ = 2.76, p = 0.005) but not ROI (F_(3,57)_ = 1.642 p = 0.19) and notably, a significant time ∗ ROI interaction (F_(27,513)_ = 1.53 p = 0.044). To determine the basis of this interaction, data from each ROI were analysed using one way repeated measures ANOVA in which the factor was time (10). The positive effect of condition was significant for all frontal lobe ROIs (Table 2) with the exception of the left IFO, which followed a sub-threshold trend in the same direction. This effect of reasoning demand was greatest in the right IFS and the left IFG. The main effects of time were also significant within the right IFS and left IFG (Table 2). Unconstrained whole brain analysis accorded well with the ROI results (Fig. 4a). Thus, when the effect of reasoning demand was examined at each time bin (FDR corrected at p < 0.05), elevated activation was evident during more challenging trials in the temporal lobes and the lateral prefrontal cortices early in the timecourse. The temporal lobe activation reduced over the first few time bins, whereas the left IFG, the right IFS and the IFO showed heightened activation throughout the first half of the timecourse. Based on these results, it was decided that activation within the right IFS during the first half of the timecourse would provide the most sensitive and selective marker of engagement in the reasoning task, a result that conforms closely with our prior imaging data (Hampshire et al., 2012).

### 3.3 Mental imagery

Brain regions that showed category specific responses to simple cues (see Methods) were used as markers of category specific imagery in response to the reasoning trials. Based on well established dissociations in terms of visual perception (Epstein and Kanwisher, 1998; Kanwisher et al., 1997) and attentional modulation (O'Craven and Kanwisher, 2000), we expected to observe heightened activation in a network of regions including the parahippocampal place area (PPA) during house imagery and in a network of regions including the fusiform face area (FFA) during face imagery. We predicted, based on the assumption that the imagery process started at around 0–2 s after the cues and continued until the command to rest, that the BOLD response should be greatest (half the peak amplitude or more) during the central 4 FIR time-bins (7–14 s). Responses to cued house imagery were contrasted with cued face imagery at these time bins and the resultant whole brain maps were analysed at the group level using a t contrast in SPM5. Heightened activation was evident for the house imagery trials bilaterally close to the parahippocampal place area (PPA) (PPA left x = − 30, y = − 42, z = − 8; PPA right x = 34, y = − 38, z = − 12, p < 0.05 FDR corrected for 2 ∗ 10 mm radii spheres around previously reported PPA locations (Hampshire et al., 2008)) and these clusters were defined as ROIs for further analysis. Surprisingly, heightened activation was not evident for cued face imagery within a 10 mm radius of the previously reported coordinates for the FFA (Kanwisher et al., 1997) even at an uncorrected threshold of p < 0.05, a result that does accord with some previous studies (Hampshire et al., 2009). However, when unconstrained whole brain analysis was carried out, extensive clusters of activation were evident in the medial frontal wall (MFW x = 4, y = 52, z = 12) and the precuneus/posterior cingulate gyrus (PC x = − 2, y = − 54, z = 28) (both p < 0.05 FWE cluster corrected). These medial brain regions have been associated with face processing in previous studies (Hampshire et al., 2008; Ishai et al., 2002) and consequently, ROIs were defined as markers of face imagery at these peak activation coordinates.

**Fig. 5:**
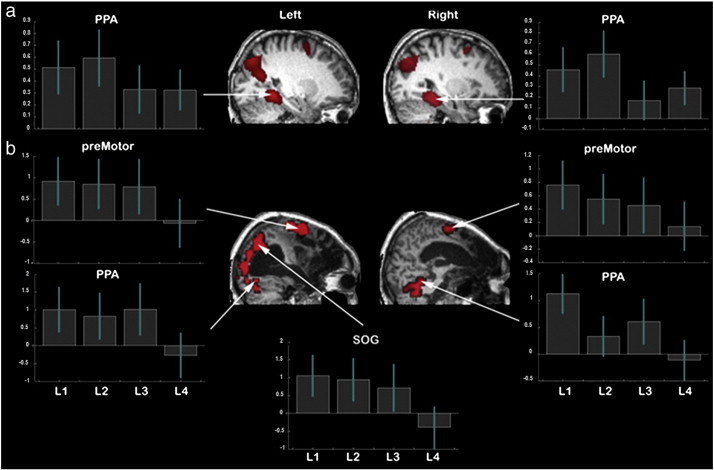
Category specific neural responses during house imagery. a — Controls showed significant activation within the parahippocampal gyrus, the superior occipital gyrus and the lateral premotor cortex bilaterally during reasoning trials on which the correct answer was house vs. those where the correct answer was face (image rendered with voxel-wise whole brain correction at p < 0.05 FDR corrected). This effect was less pronounced during more difficult reasoning trials. b — The patients showed a similar pattern of activation for the same contrast (image rendered with voxel-wise whole brain correction at p < 0.05 FDR). Notably, however, none of the category sensitive regions observed in the patient showed significant effects at the most difficult level. Error bars report the 90% confidence interval.

**Fig. 6:**
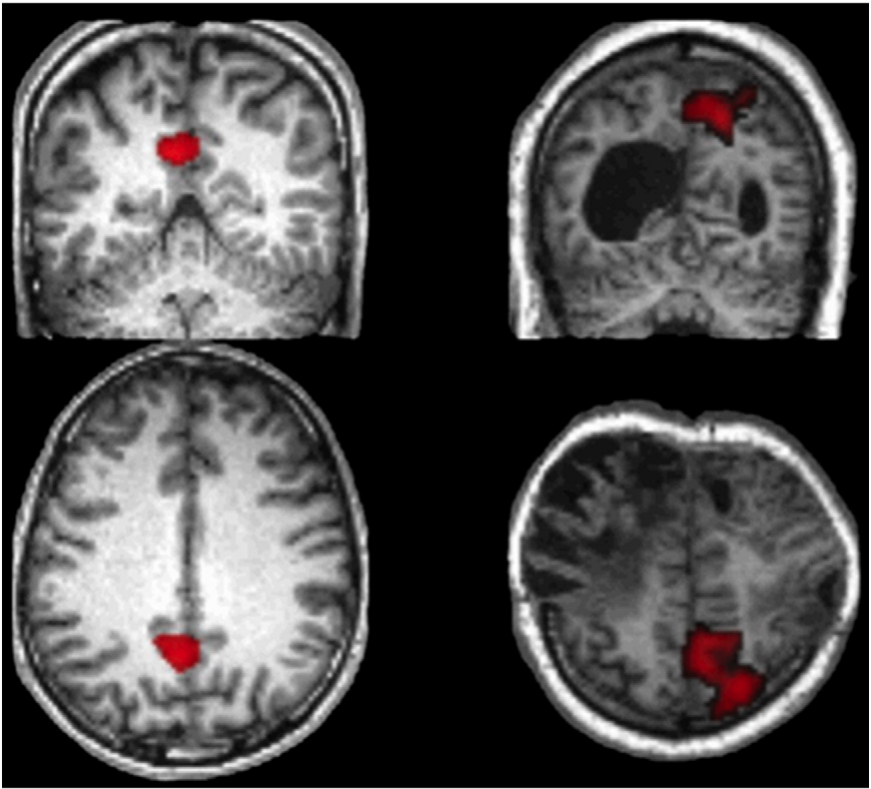
Left — In controls, contrasting reasoning trials on which the answer was face minus those on which the answer was house generated a single cluster of activation subtending the precuneus and the posterior cingulate gyrus (PC) (image thresholded at t > 2.35 and FWE cluster corrected at p < 0.05). Right — In the patient analysis, a highly similar region was evident when contrasting reasoning trials on which the answer was face minus those on which the answer was house (image thresholded at t > 2.35 and FWE cluster corrected at p < 0.05).

To determine whether participants reliably solved the reasoning problems, trials where the correct answer was face were contrasted with those where the correct answer was house and the resultant contrast images were examined at the group level using repeated-measures ANOVA with the factor time (10). The positive T-contrast (averaged across the central 4 time bins) revealed strong activation within the PPA ROIs (left t = 8.49, p < 0.001; right t = 7.65, p < 0.001). The negative T contrast generated a significant effect within the PC but not the MFW (PC t = 2.44, p < 0.01; MFW t = 1.07, p > 0.05). Similarly, the main effect of time was strong within the PPA and significant within the PC but not within the MFW (PPA left F = 10.5, p < 0.001, PPA right F = 6.75, p < 0.001, PC F = 2.76, p < 0.05, MFW F = 1.42, p > 0.05). Unconstrained whole-brain analysis (FDR correction p < 0.05) accorded well with these results (Fig. 4b). Specifically, strong PPA activation was evident mid-way through the timecourse when contrasting trials in which the correct answer was house minus those where the answer was face (left x = − 28, y = − 46, z = − 10, right x = 28, y = − 38, z = − 14). Activation was also evident in the superior occipital cortices bilaterally (left x = − 32, y = − 78, z = 32, right x = 36, y = − 78, z = 32) and the lateral premotor cortices bilaterally for this contrast (left x = − 24, y = 2, z = 60, right x = 28, y = 10, z = 54) (Fig. 5a). No activation was evident for the reverse (face minus house) contrast at the corrected threshold nor was activation evident around the expected coordinates of the FFA, even at p < 0.05 uncorrected. Instead, activity was again evident in the PC (x = − 2, y = − 56, z = 28, t = 4.05, p < 0.001 uncorrected; Fig. 6). Based on these results, it was decided that activity midway through the FIR timecourse in the PPA, the SOG, the lateral PMC, and the PC would provide the most sensitive markers of category specific imagery.

## 4 Patient neuroimaging study

### 4.1 Auditory processing

Based on the control data, signs of early auditory processing were detected using a T contrast, comparing the first 3 time bins averaged across all reasoning trials to the rest of the FIR timecourse. The results revealed two extensive clusters of activation using a family wise error (FWE) cluster correction at p < 0.05 (Fig. 2c). One cluster subtended the left posterior-superior temporal lobe and the lateral occipital lobe (1214 voxels, p < 0.001 FWE cluster corrected, peak voxel t = 5.10, p < 0.05 FDR corrected). The other subtended the right insula/frontal operculum and the most anterior remaining extent of the right dorsolateral frontal cortex (311 voxels, p < 0.005 FWE cluster corrected, peak voxel t = 3.90, p < 0.001 uncorrected).

### 4.2 Reasoning

**Fig. 7:**
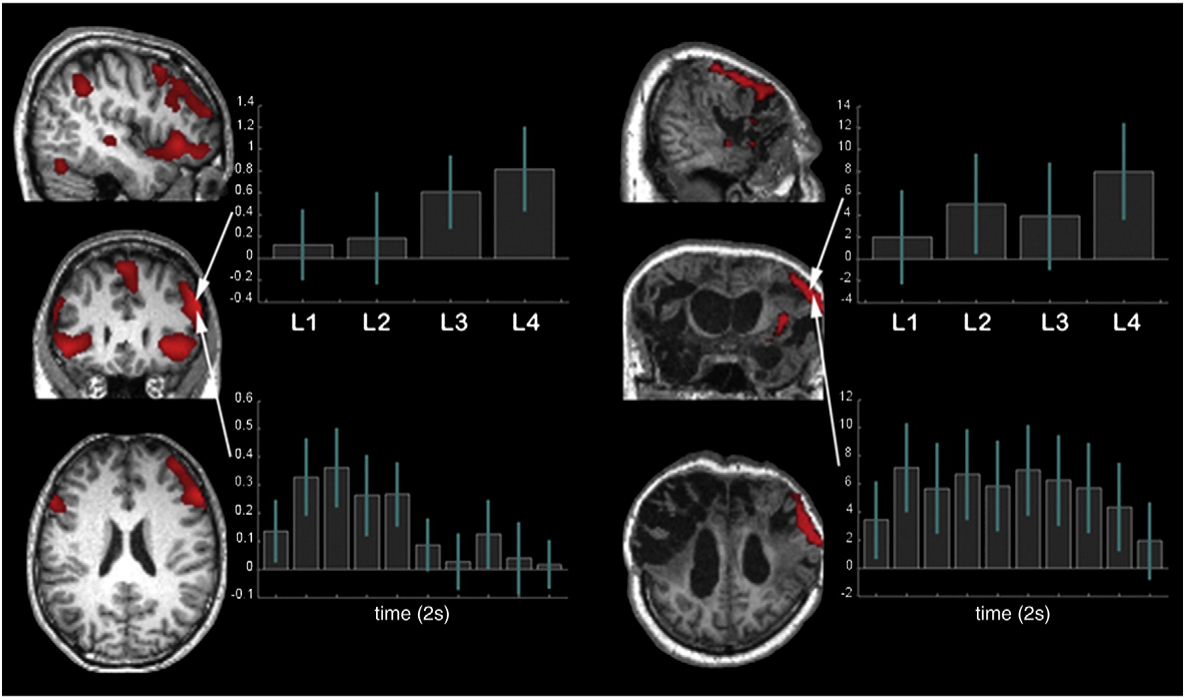
Left — In controls, the right IFS showed a particularly strong sensitivity to the level of reasoning demand. Activation within this region was predominantly evident in the first half of the timecourse. Right — When reasoning trials were contrasted with cued trials in the patient analysis, one significant cluster was evident subtending the most dorsal/anterior portion of the right cerebral cortex. An ROI at the peak activation coordinates, showed a significant sensitivity to the overall level of reasoning demand. Error bars report the 90% confidence interval.

Defining lateral frontal lobe ROIs in the patient was a particular challenge due to the extensive damage that had occurred in the more anterior and medial frontal regions. Consequently, an unconstrained voxel-wise analysis was first undertaken in which the first half of the FIR timecourse (when reasoning related activation was greatest in controls) was contrasted for all reasoning trials minus all cued trials. In close accordance with the control study, a significant cluster of activation (Fig. 7) was evident subtending much of the most dorsal and anterior portion of the right cerebral hemisphere (1037 voxels, FWE cluster corrected at p < 0.05, peak voxel t = 6.11, p < 0.001 FDR corrected). When a 10 mm radius spherical ROI was defined at the peak activation coordinates and the first half of the timecourse (when reasoning activity was greatest in controls) was examined, there was a significant effect of reasoning demand (t = 2.06, p = 0.02). More specifically, there was higher activation for the most difficult reasoning problems, a result that accords closely with the lateral frontal cortex data in controls.

### 4.3 Imagery

**Fig. 8:**
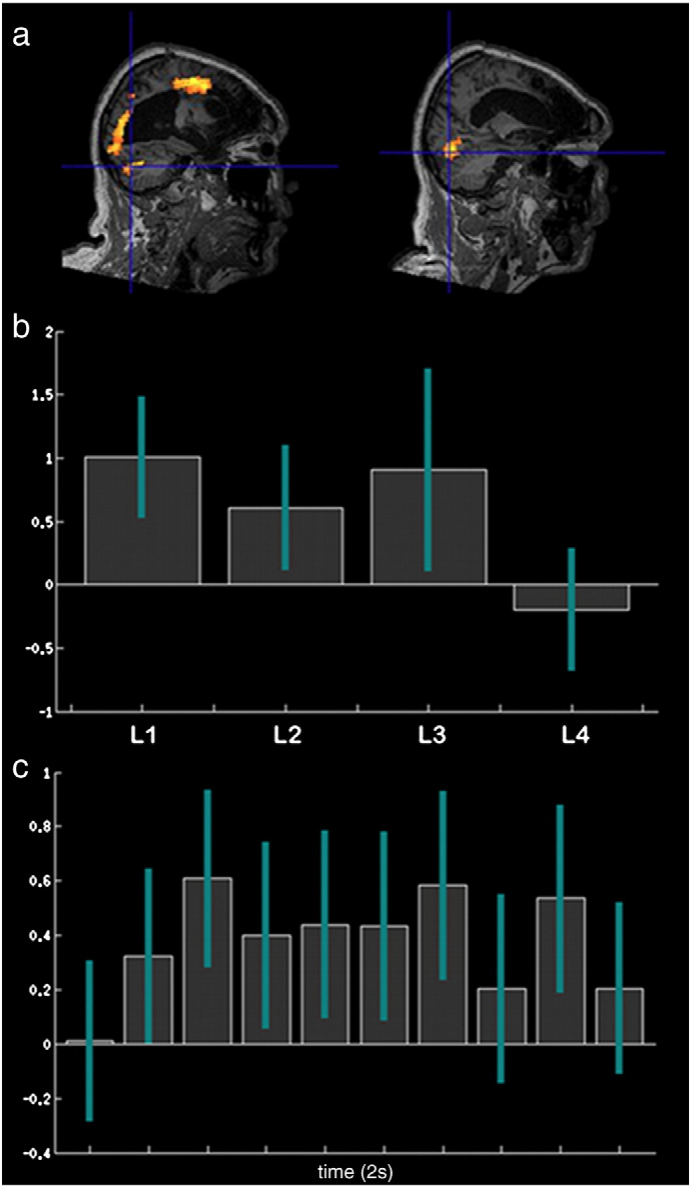
a) Contrasting house minus face imagery on cued trials generated activation close to the expected locations for the right parahippocampal place area, the left superior occipital gyrus, and the left lateral premotor cortex. b) When 5 mm radius spheres were placed at the peak coordinates and combined into a single ROI, increased activation was evident for the contrast of reasoning trials on which the answer was house minus those on which the answer was face at all but the hardest level. Error bars report the 90% confidence interval. c) The FIR timecourse for this ROI, averaged across levels, was smooth and sustained with a peak in the third time bin.

In line with the approach taken in controls, category sensitive ROIs were defined by contrasting house and face imagery in response to simple cues and these were used as markers to detect category specific imagery responses to the reasoning trials. Regions of activation were observed close to the expected location of the right PPA (t = 2.93, p < 0.005 uncorrected), the left superior occipital gyrus (t = 2.76, p < 0.005 uncorrected) and the left lateral premotor cortex (t = 4.17, p < 0.001 uncorrected) (Fig. 8). The MFW was almost entirely destroyed in this patient and there were no regions of significant activation close to the expected coordinates of the PC. 5 mm radius spheres were defined at the peak activation coordinates and combined into a single ROI. The contrast of reasoning house imagery minus reasoning face imagery was calculated for the central 4 time bins averaged across all voxels within this combined ROI. A significant effect (t = 3.32, p < 0.001) was evident, with heightened activation during trials on which the logically correct answer was the word ‘house’ vs. ‘face’. When the same contrast was examined separately at each difficulty level, there was a significant effect for all but the highest level of reasoning demand (L1 t = 3.48. p < 0.001, L2 t = 2.05, p < 0.05, L3 t = 1.87, p = 0.03, L4 t = 0.68, p = 0.75). A more conservative unconstrained voxel-wise analysis was also undertaken within the whole brain for the reasoning house–face contrast. The results confirmed those of the ROI analysis, with significant clusters of activation (peak coordinates, p < 0.05 FDR corrected for the whole brain mass) within the PPA and the lateral premotor cortices bilaterally, and within the left SOG (Fig. 5b). When the peak coordinates for these regions were examined for the reasoning house–face imagery contrast at each level of difficulty, there were again significant effects at all but the highest level of reasoning demand. Moreover, whilst a MFW ROI could not be defined from the cued trials, unconstrained whole brain analysis of the reasoning face–house contrast generated just one significant cluster that, in close accordance with the control data, was within the PC (Fig. 6) (589 voxels, FWE cluster corrected at p < 0.05, peak coordinates, FDR corrected p < 0.05).

## 5 Discussion

### 5.1 The use of frontal lobe markers for detecting engagement in the reasoning process

The results presented here, provide several new pieces of evidence to support the hypothesis that a physically non-communicative patient who is diagnosed as VS or MCS based on a purely motoric assessment, may retain an undetected capacity for higher cognitive function. The observed response of the right IFS during reasoning trials is of particular note, because it provides a neural marker of engagement in the reasoning process. More specifically, in controls, this area responded selectively during reasoning trials, showed a strong increase in activity across the 4 levels of reasoning difficulty and was transiently active towards the earliest stage of the trial, when reasoning occurred. The observed selectivity to the reasoning demand of the IFS (often labelled the mid-dorsolateral prefrontal cortex (Petrides, 1994)), accords particularly well with previous studies, in which it has been demonstrated that the IFS tends to be recruited in response to more complex (Owen et al., 1996), although not necessarily more difficult (Bor et al., 2001) cognitive demands. By contrast, the IFO and IFG tend to be recruited more generally whenever attention or wilful control is applied (Hampshire et al., 2008, 2010; Owen and Hampshire, 2009), an observation that is also supported in the current study as these regions showed sustained activation at all stages of the task and at all levels of reasoning demand. Indeed, similar IFS coordinates were shown to be sensitive to the complexity of rules during non-verbal reasoning when controlling for general difficulty effects using an orthogonal manipulation (Hampshire et al., 2011). Furthermore, in a recent study, we report activation at these exact coordinates for a broad range of tasks in which information is transformed in the mind, including grammatical reasoning, deductive reasoning, spatial rotation, and spatial planning (Hampshire et al., 2012), but little involvement in simpler paradigms, for example target detection (Hampshire et al., 2009) and working-memory maintenance. Taken together, these results reinforce the view that the right IFS is selectively recruited during the transformation of information according to logical rules (Hampshire and Owen, 2010). It is likely, therefore, that the patient engaged in the reasoning task, bringing those frontal-lobe resources that were still available on-line in response to more complex problems. This view is supported further, by the fact that, in close accordance with the control data, activation within the same area was also correlated with the overall level of reasoning demand in the control and the patient studies.

### 5.2 The use of mental imagery markers for assessing reasoning aptitude

More importantly, the evidence for frontal lobe engagement in the reasoning process is corroborated by the fact that activation was observed within the correct category specific brain regions in response to all but the most difficult reasoning problems. These imagery responses provide a stronger indicator of higher cognitive function because the pattern of activation in response to reasoning problems not only matched that observed in controls, but also cross-validated against another contrast within the participant's own data set. More specifically, in order to communicate his answer, the patient had to signal his response by deliberately modulating activity within the same network that he had previously activated in response to simple cued imagery trials. Thus, the imagery responses form a neural analogue of the button response used to signal responses in standard cognitive testing batteries. It should also be noted, that the lack of a significant effect at the highest level of reasoning demand in the patient study also makes intuitive sense, as it is unlikely that an individual would be able to solve difficult problems whilst failing easy problems. Moreover, given the extensive damage to the frontal lobes, it would be particularly surprising if the patient were reliably able to solve the most difficult problems, as approximately 1/7 of the healthy young controls performed this level at chance in the behavioural pilot study. Taken together, these results accord closely with the view that the patient was able to process reasonably complex verbal statements and to infer logical answers. Indeed, to successfully perform the task, it was necessary to maintain the rules that make up the overarching task set, maintain the focus of attention, process auditory stimuli, logically transform the content of complex verbal statements, and engage in mental imagery.

### 5.3 Ethical implications

Beyond a better characterisation of cognition in a physically non-communicative patient, the results presented here have some bearing on important ethical issues. Given the current drive towards developing modes of communication for patients who are overtly non-communicative, the question will inevitably arise, regarding the level of control one should give an individual over decisions regarding their long-term care. To address this issue, it will be critical to determine whether an individual is able to understand complex questions and make logical inferences. This is a complicated problem for any cognitively impaired individual; however, the lack of an overt means of response makes this a particularly intractable issue with VS and MCS patients. We suggest that the design presented here could be readily adapted to assess a broad range of cognitive abilities and consequently, the development of a cognitive assessment battery is likely to be tractable using this approach. To this end, a number of improvements to the current design could be considered in future studies.

### 5.4 Proposed design improvements

The category specific response during the imagery of faces was not as robust as that observed for houses. More specifically, whilst PC activity did provide a successful marker of face imagery in controls and patients, it was statistically weaker and moreover, FFA activity was not evident at all. This latter point is interesting in its own right as it seems somewhat incongruent with the strong prior literature reporting category specific attentional modulation in this brain region (O'Craven and Kanwisher, 2000). One possibility is that the FFA is only engaged during the visual perception of, but not the mental imagery of faces, with the overall level of activation being modulated as a function of attention only when a face is present in the visual field. Notably, however, the results do accord with the previous observation, that whilst top-down attentional manipulations targeted at differentially modulating the face or building component within overlapping/compound stimuli have a significant effect on activity within the PPA, they are harder to detect within the FFA (Hampshire et al., 2009). It has previously been suggested that the FFA may be specialised for processing information for which people are expert as opposed to faces in particular (Gauthier et al., 2000). This suggestion could also account for the lack of FFA activation in the current design, as participants were asked to imagine either the face of a family member or different views of a familiar building, both items for which they would be expert. Either way, in future studies, a sensible alternative, could be to replace face imagery altogether, perhaps with motor imagery, which has proven to be reliable in previous studies with VS patients (Monti et al., 2010; Owen et al., 2006).

A sensible future direction would be to develop a real-time version of the task (Cusack et al., 2012) that dynamically varies the length of the imagery time-period based on a certainty criteria and that increments difficulty based on evidence of success. This more interactive design would be closer to what is generally meant by a ‘brain computer interface’ as it would interact and adapt the task parameters in response to changes in brain activity. This alteration could also provide a finer grained assessment of an individual's reasoning ability.

Finally, it has been suggested that EEG is a more practical approach when testing severely motor impaired patients, as portability of the hardware allows the experiment to be carried out at the bedside (Cruse et al., 2011). Consequently, development of an EEG analogue of this paradigm could provide a more practical tool for assessing higher cognitive function in individual patients. It should be noted though, that the higher spatial resolution of fMRI makes it more suitable for concurrently examining the neural circuits that contribute to the reasoning process. Indeed, the concurrent observation of increased right IFS activation at greater reasoning demand effectively cross-validates the analyses of mental imagery responses. Furthermore, the combination of the slow BOLD response with a flexible FIR model, provides a measure of functional brain activity that is simple and robust against confounding factors like cognitive slowing (Sawamoto et al., 2002) that may uncouple the timing of cognitive events from the neural response in VS and MCS patients (Bardin et al., 2011).

### 5.5 Summary

In summary, the current study provides several cross-validating pieces of evidence to support the view that a patient with a VS or MCS diagnosis may retain the capacity to solve logically complex verbal problems. Thus, there was temporal lobe activation in response to the auditory stimuli, lateral frontal cortex activation in response to reasoning problems that scaled with demand, and activation within the category specific regions that corresponded to the item that formed the logically correct answer. It is important to note that some patients might not be able to engage in mental imagery and therefore, their reasoning ability could not be assessed in the manner reported here. Similarly, some previously reported paradigms are likely to be more sensitive for detecting mental imagery in those individuals who have a reasoning deficit. Consequently, we suggest that the type of method reported here, would most sensibly be applied as part of a second level assessment. That is, when trying to gauge higher cognitive abilities in those patients who have already been observed to engage in mental imagery in response to simple auditory instructions. Future studies should focus on optimising the design, adapting it to assess a broader range of cognitive abilities, and determining the proportion of VS and MCS patients who retain such abilities.

The following are the supplementary data related to this article.

Supplementary Table 1 Medications.

Supplementary Table 2 Neurologist reports.

Supplementary data to this article can be found online at http://dx.doi.org/10.1016/j.nicl.2012.11.008.

## Figures and Tables

**Table 1:** Statements.

Statement	Level	Type
The house precedes the face	L1	Active/positive
The house follows the face	L1	Active/positive
The house is followed by the face	L2	Passive/positive
House is preceded by the face	L2	Passive/positive
House does not precede face	L3	Active/negative
House does not follow face	L3	Active/negative
House is not followed by the face	L4	Passive/negative
House is not preceded by face	L4	Passive/negative

**Table 2:** Effect of difficulty level in the frontal lobe ROIs.

Contrast	ROI	Con	t	p corrected
Positive effect of condition	Left IFO	0.76	2.17	0.061
	Right IFO	1.07	2.97	**0.007**
	Right IFS	1.65	4.34	**< 0.001**
	Left IFG	1.29	3.46	**0.001**

Contrast	ROI	Con	F	p corrected

Main effect of time	Left IFO	2.05	2.07	0.13
	Right IFO	2.46	2.38	0.058
	Right IFS	4.05	3.60	**0.001**
	Left IFG	4.36	4.07	**< 0.001**

Numbers in bold are significant at the p < 0.05 threshold.

**Table 3:** Patient behavioural assessments.

Visit	Interval (months)	Assessment day	Coma recovery scale						
			Total	Auditory	Visual	Motor	Oromotor/verbal	Communication	Arousal
1	18	Max of 4^a^	6	1 — startle	0 — none	2 — flexion withdrawal	1 — oral reflexive	0 — none	2 — eyes open w/o stimulation
2	30	1	5	1 — startle	1 — startle	1 — abnormal posturing	0 — none	0 — none	2 — eyes open w/o stimulation
		2	3	1 — startle	1 — startle	1 — abnormal posturing	0 — None	0 — none	0 — none
		3	2	0 — none	0 — none	2 — flexion withdrawal	0 — none	0 — none	0 — none
		4	10 (MCS +)	3^b^	3 — pursuit	2 — flexion withdrawal	0 — none	0 — none	2 — eyes open w/o stimulation
3	36	1	5	1 — startle	1 — Startle	2 — flexion withdrawal	0 — none	0 — none	1 — eyes open with stimulation
		2	7 (MCS-)	1 — startle	3 — pursuit	2 — flexion Withdrawal	0 — none	0 — none	1 — eyes open with stimulation
4	54	1	6	1 — startle	1 — startle	2 — flexion withdrawal	1 — oral reflexive	0 — none	1 — eyes open with stimulation

a At this time only the maximum score from all 4 days was recorded.

b Moved but did not stop on command.
