# Supplementary material for: Assessing residual reasoning ability in overtly non-communicative patients using fMRI
Source: Neuroimage Clin. 2012 Nov 30;2:174–83. doi: 10.1016/j.nicl.2012.11.008 (PMC3777757; doi:10.1016/j.nicl.2012.11.008)
Supplement: Supplementary Table 1 — Medications. [file mmc1.docx]

|  | Assessment 1 | Assessment 2 | Assessment 3 |
| --- | --- | --- | --- |
| Medications | Baclofen (30mg tds) | Baclofen (30mg tds) | Baclofen (30mg tds) |
|  | Gabapentin (500mg tds) | Gabapentin (500mg tds) | Gabapentin (500mg tds) |
|  | Dantrolene (50mg qds) | Dantrolene (50mg qds) | Dantrolene (50mg qds) |
|  | Carbamazepine (200mg od) | Carbamazepine (200mg od) | Carbamazepine (200mg od) |
|  | Domperidone (10mg tds) | Domperidone (10mg tds) | Domperidone (10mg tds) |
|  | Omeprazole (40mg od) | Omeprazole (40mg od) | Omeprazole (40mg od) |
|  | Enoxaparin (40mg s/c od) | Diazepam (10mg prn) | Diazepam (10mg prn) |
|  |  |  | Phenytoin (300mg) |
|  |  |  | Clarithromycin (500mg bd) |
