# Supplementary material for: Assessing residual reasoning ability in overtly non-communicative patients using fMRI
Source: Neuroimage Clin. 2012 Nov 30;2:174–83. doi: 10.1016/j.nicl.2012.11.008 (PMC3777757; doi:10.1016/j.nicl.2012.11.008)
Supplement: Supplementary Table 2 — Neurologist reports. [file mmc2.docx]

| Visit | Neurologist report |
| --- | --- |
| 1 | The patient has spontaneous eye-opening, a repertoire of reflexive behaviours, and no signs of awareness of himself nor his environment |
| 2 | He had a regular breathing pattern with a respiratory rate of 16 / min., pulse was 75 and regular and BP 125/72.  Pupils were equal and slowly responsive to direct and indirect light. Increased lacrimation was noted after examination on one occasion. He had normal Dolls eye response, bilaterally.  He had a brisk pout reflex.  There were occasional asymmetrical grimaces (with most movements on the right side of the face) but these were not provoked consistently in response to painful stimuli such as deep nail bed pressure.  His muscle tone relaxed. Reflexes were absent except at the left biceps, bilateral finger jerks and the plantar responses was flat on the left and variably flexor on the right. He had a regular breathing pattern with a respiratory rate of 16 / min., pulse was 75 and regular and BP 125/72.  Pupils were equal and slowly responsive to direct and indirect light. Increased lacrimation was noted after examination on one occasion. He had normal Dolls eye response, bilaterally.  He had a brisk pout reflex.  There were occasional asymmetrical grimaces (with most movements on the right side of the face) but these were not provoked consistently in response to painful stimuli such as deep nail bed pressure.  His muscle tone relaxed. Reflexes were absent except at the left biceps, bilateral finger jerks and the plantar responses was flat on the left and variably flexor on the right. |
| 3 | For most of the admission the patient had a regular breathing pattern with a rate of 16 / minute. His pulse was 75 and regular and he had a BP of 125/72. He had a large bilateral frontal cranial defect with deep frontal recession and lateral bulging of soft tissue over the rim of the carnioplasty.  Pupils were equal and slowly responsive to direct and indirect light. He had a normal Dolls eye response, bilaterally.  Reflexes were reduced bilaterally but present in all limbs, plantar responses were flexor. |
